# Efficacy of artemether-lumefantrine and artesunate-amodiaquine as first line therapy of uncomplicated malaria in Burkina Faso, 11 years after policy change

**DOI:** 10.11604/pamj.2020.35.68.20849

**Published:** 2020-03-10

**Authors:** Issaka Zongo, Yves Daniel Compaoré, Frédéric Nikiéma, Moussa Zongo, Nouhoun Barry, Fabrice Anyirékun Somé, Naomie Kaboré, Jean Bosco Ouédraogo

**Affiliations:** 1 Institut de Recherche en Sciences de la Santé, Direction Régionale de l'Ouest, Bobo-Dioulasso, Burkina Faso

**Keywords:** Burkina Faso, first line ACTs efficacy, malaria

## Abstract

**Introduction:**

Artemether-lumefantrine (AL) and artesunate-amodiaquine (ASAQ) are the first line therapy of uncomplicated malaria in Burkina Faso. We assessed the treatment efficacy, tolerability of these drugs 11 years following its adoption as first line treatment.

**Methods:**

In this opened randomized controlled trial carried out in 2016, participants with age over 6 months who consented to participate were randomly assigned treatment with artemether-lumefantrine or artesunate-amodiaquine and followed up for 28 days. Primary endpoint was the treatment efficacy over 28 days of follow up unadjusted by Polymerase chain reaction (PCR).

**Results:**

Two hundred and eighty-one (281) participants were enrolled and the completion rate was 92.9%. No early treatment failure was found. Adequate clinical and parasitological responses were significantly higher in artesunate-amodiaquine group (97% versus 85.2%, p = 0.0008). On day 28, the risk of failure was 4 times higher in AL group 20.14%, 95% CI (13-30.47) against 5.16%, 95% CI (1.91-13.54) in ASAQ group. All treatments had a similar and good tolerability profile.

**Conclusion:**

Eleven years following artemether-lumefantrine and artesunate-amodiaquine adoption as first line therapy for uncomplicated malaria in Burkina Faso, artemether-lumefantrine retained fairly good efficacy even though its efficacy fell below WHO threshold of 90% considering uncorrected outcome.

## Introduction

The cornestone of malaria treatment has been since the last fithteen years the artemisinin based combination therapy (ACTs) mainly artemether-lumefantrine and artesunate-amodiaquine. Procurement of ACTs faced severe shorted during the first five to ten years of its adoption. Currently, the procurement market has improved and ACTs are now widely distributed. Over 176 metrics tons of ACTs have been dispensed in endemic sub-Saharan Africa and Asia in 2017 and this demand is expected to growth up to 196 tons metrics in 2018, 205 MT in 2019 and 221 MT in 2020 [1]. Efficacy studies have demonstrated the outstanding efficacy of these drugs in the treatment of uncomplicated malaria in either Africa [2-4] or Asia [5]. Effectiveness studies after its introduction in the malaria fight tools have reported a sustained efficacy in different countries with different epidemiological patterns [6, 7]. However, despite the implementation of these effective tools (chimiotherapy, chemoprevention, LLINs), malaria remains proeminent in sub-Saharan Africa and particularly in Burkina Faso. Despite success gained through multiple interventions strategies, malaria continues to weight a high burden in the world's health [8, 9] and Burkina is one of the 10+1 country contributors [10]. This situation inevitably sustains the use of the ACTs and thus the increase in drug pressure to the used drugs (artemether-lumefantrine and artesunate-amodiaquine). As drug pressure is known to contribute to the alteration of drug efficacy [11] , this continuous ACT use over 11 years warrants the investigation of this first line therapy efficacy; the present report is intented to provide updated efficacy data for artemether-lumefantrine and artesunate-amodiaquine in Burkina Faso to guide the national malaria control program.

## Methods

**Study site:** the study was carried out in two peripheral health facilities, Colsama and Sakaby during the transmission season between June and December 2016. Each health facility was staffed by two to three nurses and one midwife. They were equipped with one outpatient department and one inpatient wall. All sites were located in Bobo-Dioulasso the second capital city of Burkina Faso at 365 km from Ouagadougou. Transmission of malaria is long but seasonal occurring over 6 months and containing at least 60-80% of clinical cases.

**Study participants:** subjects presenting to the study health facilities with fever (axillary temperature ≥ 37.5°C) or history of fever in the previous 24 hours were screened against the following inclusion criterias in accordance with WHO guideline 2015: age at least 6 months, weight at least 5 kg, no evidence of concomitant illness, the provision of informed consent by the parents and the ability to participate in 28 days follow up, no history of antimalarial treatment within the last two weeks, no danger sign or evidence of severe malaria, P.falciparum mono infection, parasite density of 2000-200 000 parasites per µl of blood and hemoglobin ≥ 5 g/dL. During the screening process, participants satisfying the above criterias were enrolled while those who were excluded were referred to the health facility staff for standard care.

**Evaluation at entry and randomization:** potential participants were assigned a number (ID), interviewed on past medical history, examined and then referred to the study nurses dedicated only to treatment allocation. Participants were randomly allocated to oral three days treatments based on a computer generated code provided by a staff not involving in the patient's evaluation. The treatment was either artemether-lumefantrine or artesunate-amodiaquine. The dosage was given on weight basis as per national malaria control program recommendation as this study is intented to evaluate the efficacy of first line treatments of malaria as implemented by the national malaria control program: (a) Artemether-lumefantrine (Coartem^®^ Novartis administrated in tablet containing 20 mg of artemether plus 120 mg of lumefantrine); (b) artesunate-amodiaquine as given as tablet containing 67.5 mg plus 25mg respectively). The study as per its objectives is to inform straight away, it was not blinded to the study staff and patient. The treatment was administrated as DOT (directly observed treatment) and participants were retained for 30 minutes and in case of vomiting within this time period, a replacement dose was given. Any repeated vomiting led to the participant exclusion from further follow up and referral to health facility staff for standard care. Children presenting with hemoglobin less than 10 g/dL were treated according to the Integrated management of childhood Illness guidelines with ferrous sulfate for 14 days and anthelmintic treatment, if appropriate.

**Follow up and classification of treatment outcome:** patients were asked to return to the study clinics on day 1,2,3,7,14,21 & 28 and at any time they were feel illness. Field workers visited those who failed to return to the clinics and each visit consisted on standard case report completion, physical examination, a finger pick for thin and thick blood smear. Blood smears were read to assess the parasite density and gametocytes. Patients were followed up for 28 days and their outcomes were assessed according to World Health Organisation (WHO) guidelines for antimalarial treatment efficacy [12]. The outcome was classified as: early treatment failure (presence of danger signs, complicated malaria, or failure to adequately respond to therapy within first three days), late clinical failure (presence of danger signs, complicated malaria or fever with parasitemia during day 4-28), late parasitological failure (presence of parasitemia without clinical findings during days 7-28), or adequate clinical and parasitological response (absence of parasitemia through follow up). Secondary outcomes included the resolution of fever, parasite clearance, change in hemoglobin, presence of gametocytes during follow up and the occurence of adverse events. Alternative treatment for those who failed treatment was quinine (10mg per body weight per day for 7 days). Patients were excluded from further follow up for antimalarial use outside the study, lost to follow up, informed consent withdrawal or serious adverse event.

**Laboratory procedures:** blood smears were stained with 2% Giemsa for 30mn. Parasites density was determined after thick film reading through microscope and calculated as the number of asexual parasites per 200 leucocytes (or per 500 leucocytes if the number of asexual parasites were less than 10 per 200 leucocytes) assuming a leucocyte count for 8000 leucocytes per microliter. Smears were considered to be negative if microscopic examination of 100 high power fields did not reveal any parasite. Reading was done by two expert microscopists and reconciliation by a third one in case of results discrepancies (difference between two readers more than 25%). Gametocytes were recorded as present or absent. Hemoglobin level was measured from finger prick blood sample using a portable photo spectrophotometer (HemoCue).

**Statistical analysis:** the study was designed to check the efficacy of first line treatment of malaria in Burkina Faso, artemetgher-lumefantrin and artesunate-amodiaquine. We anticipated that 136 participants will be needed in each group to assess the efficacy of artemether-lumefantrin versus artesunate-amodiaquine assuming 90% uncorrected cure rate in artesunate-amodiaquine group (detecting a difference of 15%) with 80% and significance level of 0.05 accounting for 20% of lost to follow up. Baseline characteristics of enrolled participants will be summarized; participants in each category (anemic or not) will be presented as proportion and compared between drug regimen. Categorical variables will be compared dosing chi square test while continuous are compared using either independent or dependant t-test. Kaplan Meyer estimate will be used to calculate the risk of treatment failure using strick formula and patient were censored the day they left the study.

**Ethics approval and consent to participate:** the study was conducted as part of routine National Malaria Control Program monitoring and evaluation of first line therapies efficacy. It approved by the Institutional Ethics Committee for Health Research of the Institut de Recherche en Sciences de la Santé. Written informed consents were obtained from all parents or guardians of children who participated in the study. Participants were explaining the full scoop of the study and their right to participate or not without any prejudice; participation could be stopped at any time without any explanation. Subjects with malaria were treated in accordance with the National Malaria Control Program guideline.

## Results

**Figure 1:**
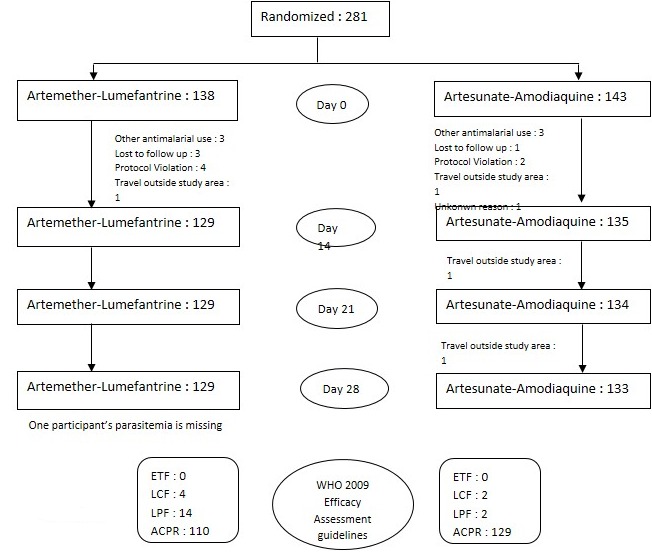
Trial profile: this figure shows the number of participants of the study at different time point starting from enrollment to the last day of follow up in each arm; overall, more than 90% of enrolled participants finished the study and have an assigned outcome

**Baseline characteristics of enrolled participants:** a total of 281 children were enrolled over the study period and treated with either AL or ASAQ for three days. About 43% of the participants treated with AL and 56.7% of those treated with ASAQ were male. Children 5-9 years were more represented. Mean hemoglobin was 10.82±1,2 g/dL and 10.92±1,85 g/DL respectively in AL and ASAQ groups. Other characteristics are described in Table 1 and Figure 1.

**Table 1:** Baseline characteristics of enrolled participants

Patients’ parameters	Artemether-lumefantrine	Artesunate-Amodiaquine
Enrolled	138	143
Gender, male (%)	55 (43%)	72 (56.7%)
Age		
Mean (SD)	9.85 (10.21)	9.42 (8.33)
Median	7	7
6-11 months	1 (0.72%)	1 (0.7%)
12-23 months	7 (5.07%)	11 (7.69%)
24-59 months	30 (21.74%)	23 (16.08%)
5 -9 years	55 (39.86%)	60 (42.96%)
10-14 years	24 (17.39%)	32 (22.38%)
Over 14 years	21 (15.22%)	16 (11.19%)
Mean temperature, °C (SD)	38.5°C (1.15)	38.5°C (1.22)
Mean weight, kg (SD)	27.75 (20.4)	26.35 (16.20)
Geometric mean of parasitemia (95%CI)	27572 [22332-34040]	31401 [25580-38547]
Mean haemoglobin, g/dL (SD)	10.73 [10.47-11.00]	10.72 [10.44-10.99]

**Treatment efficacy:** we did not record any early treatment failure over the 28 days follow up period in all treatment groups. Late clinical failures were recorded in all groups: 5 cases 3.9% in AL and 1.5% in ASAQ group meaning that patients treated with AL were 2.6 more likely to have late clinical failure compared to patients treated with ASAQ (p = 0.2). All cases were above 59 months except the failure in the AL group which occurred in 16 months old boy. On the other hand, late parasitological failures were more common, 10.9% and 1.5% respectively in AL and ASAQ groups; patients treated with AL were almost 8 times likely to experience late parasitological failure than those treated with ASAQ (p = 0.0016). Finally, treatment was significantly successful in ASAQ group (Table 2) compared to AL group (97% versus 85.2%, p=0.0008).

**Table 2:** Treatment outcomes (WHO 2009 classification)

	Artemether-lumefantrine (Coartem^®^)	Artesunate-Amodiaquine (Coarsucam^®^)	p-value
Early treatment failure (ETP)	0	0	
Late clinical failure (LCF)	5 (3.9%)	2 (1.5%)	0.2
Late parasitological failure (LPF)	14 (10.8%)	2 (1.5%)	0.0016
Adequate clinical and parasitological response (ACPR)	109 (85.2%)	129 (97%)	0.0008

**Figure 2:**
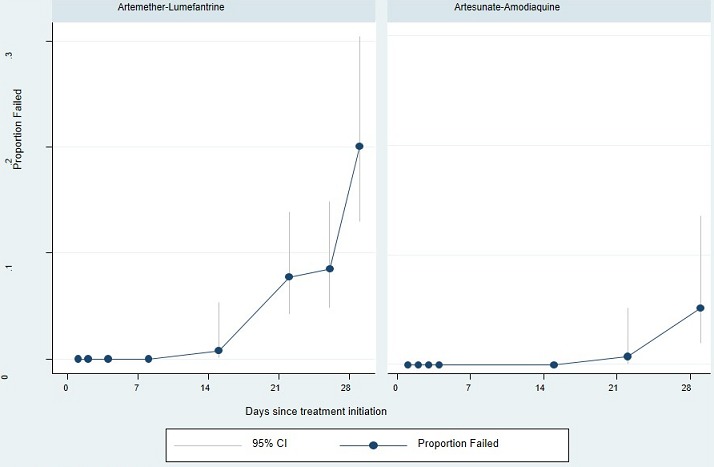
Kaplan Meier failure function: this figure shows the risk of treatment failure at each time point in the follow up and when participant left and were censored; the cumulative number of failures increased gradually in artmether-lumefantrine group while in the artesunate-amodiaquine group, the line remained on zero up to day 14

**Risk of treatment failure:** the risk of treatment failure expressed using Kaplan Meier failure function analysis revealed that this risk was null on day 7 in all treatment groups; this risk raised up to 0.78%, 95%CI (0.11%-5.37%) on day 14 with AL but remains null for ASAQ. Three weeks after the treatment initiation (day 21), the risk of treatment failure reached 7.75%, 95% CI [4.25%-13.93%] in AL group against 0.75%, 95% CI [0.11%-5.2%] in ASAQ group. On day 28, the risk of failure was 4 times higher in AL group 20.14%, 95% CI (13%-30.47%] against 5.16%, 95% CI (1.91%-13.54%) in ASAQ group (Table 3 and Figure 2).

**Table 3:** Risk of treatment failure over 28 days follow up

	Artemether-lumefantrine	Artesunate-Amodiaquine	p-value
Day 7	0	0	-
Day 14	0.78%, 95% CI [0.11 to 5.37]	0	-
Day 21	7.75%, 95% CI [4.25 to 13.93]	0.75%, 95% CI [0.11 to 5.20]	0.001
Day 28	20.14%, 95% CI [13 to 30.24]	5.16%, 95% CI [1.91 to 13.54]	0.008

**Clinical symptoms:** clinical symptoms were common at entry for all two drugs regimen. Weakness was observed in approximately 58% of participants in each arm but vomiting was more frequently observed with ASAQ (78.83% versus 65.94%, p = 0.1) (Table 4).

**Table 4:** Clinical symptoms at entry

Symptoms, % (n/N)	Artemether-lumefantrine	Artesunate-Amodiaquine	p-value
Weakness	57.97% (80/138)	58.04% (83/143)	0.99
Diarrhea	11.59% (16/138)	11.89% (17/143)	0.9
Vomiting	65.94% (91/138)	78.83% (107/143)	0.1
Headache	84.87% (101/119)	87.8% (108/123)	0.6
Abdomen pain	95.41% (104/119)	82.11% (101/123)	0.2
Pruritis	1.45% (2/138)	3.5% (5/143)	0.2
Nausea	13.45% (16/119)	22.58% (28/124)	0.06
Cough	32.61% (45/138)	38.46% (55/143)	0.3

**Treatment tolerability:** over the study period, we found no serious adverse event. The registered adverse events were mild to moderate and none required active treatment or intervention's discontinuation. Common adverse events were weakness in either group. No pruritus or nausea were found (Table 5).

**Table 5:** Prevalence of adverse events

Adverse events, % (n/N)	Artemether-lumefantrine	Artesunate-Amodiaquine	p-value
Weakness	6.25% (5/80)	6% (5/83)	0.9
Diarrhea	6.25% (1/16)	0	-
Vomiting	0	0.9% (1/106)	-
Headache	1% (1/100)	0.9% (1/108)	0.9
Abdomen pain	0.9% (1/104)	0.9% (1/101)	0.9
Pruritis	0	0	-
Nausea	0	0	-

**Figure 3:**
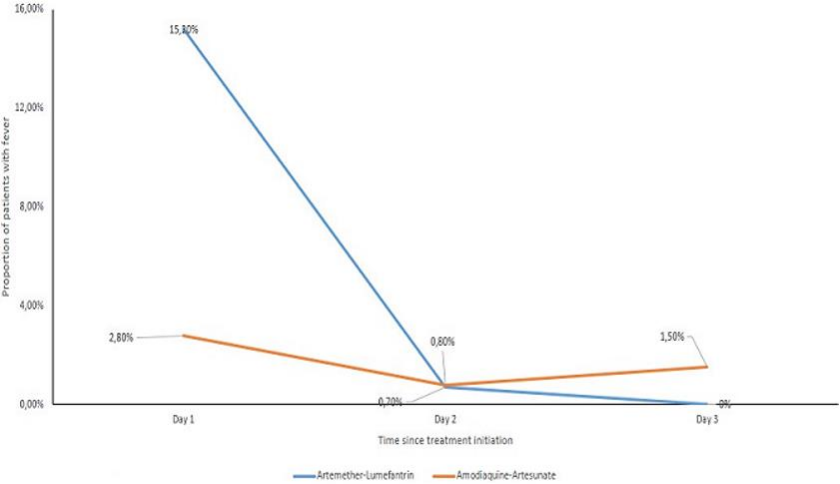
Fever clearance time: this figures shows the trend in fever relief following the initiation of the treatment in each arm; almost all patients were apyretic on day 2 and 3 for artemether-lumefantrine treated participants and very few remained on day 3

**Fever and parasite clearance time:** over 48 hours after treatment initiation, almost all participants cleared fever with only 1 fever case in each group. On day 3, no participant had fever in AL group. Two rather isolated fever without parasitemia were notified in ASAQ group (No fever on previous and following day) (Figure 3). AL treated participants achieved full parasite clearance over the first 3 days. We found 4 participants with low parasitemia in ASAQ group (1 participant with 160 parasites per microliter and 3 participants with 80 parasites per microliter).

## Discussion

This clinical trial was a study that is part of routine monitoring of first line therapies for uncomplicated malaria in Burkina Faso as per National Malaria Control Program recommendation. Eleven years following its introduction as first line therapies for malaria, AL and ASAQ remain effective in Burkina Faso even if efficacy of AL based on PCR uncorrected results was below 90% probably related with the fact that AL administration was done without fat food. Importantly, there is no early treatment failure or failures up to day 7; this reveled a good evidence that ACTs still work very well in this part of Africa and that currently the risk of tolerance to these first lines ACTs is limited. Also, all three daily doses were fully supervised by study team and a replacement was given once in case of vomiting. Previous studies in research settings have already reported good efficacy of AL and ASAQ in the central part of Burkina Faso [13], but also in other parts of the continent and Asia [2, 4, 5, 14-16]. Historically, drug resistance translated into treatment failures started in South East Asia, then migrates slowly to reach East Africa before West African region [17]. Furthermore, the mutation K13 conferring mutation to artemisinin drugs are not yet found in neighboring countries [18] even though mutations not involved in the resistance were already found [19]. Thus, we can expect these drugs to retain some efficacy for a while. In this study, all treatments doses were directly observed and this may have contributed to reach such high efficacy level.

All treatment failures recorded were late after day 14. Late failures are mainly related to which extend the partner drug lasted in the bloodstream. Despite the absence of PCR correction, the relative short half-life of lumefantrine [20, 21] and amodiaquine [22-24] were likely to explain treatment failures due to both reinfection and late recrudescence as reported in this paper. Treatments were well tolerated as seen in clinical efficacy trials across the endemic settings [25-30]. No serious adverse event was recorded in this study. This study was limited by the lack of PCR correction to distinguish true recrudescence with new infection and the limited follow up period of 28 days for these ACTs; nonetheless, the results obtained are of great relevance since it described the real time behavior of the drugs in term of efficacy and tolerability and the late occurrence of the failures were in line with clinical trial previously reports; the time limit of follow up to 28 days may have contributed to underestimate the failures but has the advantage to reduce the risk of new infection. More generally, these ACTs (AL and ASAQ) retained a certain efficacy in the treatment of uncomplicated malaria; however, some studies have reported an efficacy level of AL during 28 days follow up below the threshold set for the ACTs adopted [7,31]; for all these studies, PCR corrected outcomes were above 90% success. More than scientific interest or intrinsic efficacy of the drugs, it is questionable to whether ACTs real life efficacy should be based on the PCR corrected results or the uncorrected one or a combination of the two approaches. It may be time to initiate the discussion on current first line drugs replacement and investigate to which extend retrieved drugs (non ACT drugs like chloroquine) could be given a new life for the treatment of malaria [32].

## Conclusion

This study completed in Burkina Faso as part of the regular monitoring of first line therapies of malaria has provided some up to date efficacy data for AL and ASAQ. The drugs retained a certain efficacy with good safety profile throughout follow up period. As of other studies, AL uncorrected results fall below WHO's threshold and merit more discussion to how to react in the context of limited available ACTs and the possibility to give a new life to some old drugs.

### What is known about this topic

- Artemether-lumefantrine and Artesunate-Amodiaquine are first line therapies for malaria treatment in most countries and in Burkina Faso particularly;
- The apparition of resistant strains stressed the monitoring of drug efficacy.

### What this study adds

- Overall these drugs remain effective even though the uncorrected efficacy is below the threshold of more than 90% recommended by the WHO;
- This study rises the discussion around the Polymerase Chain Reaction correction to consider the drug efficacy in real life.

## Competing interests

The authors declare no competing interests.
